# Radioresistance in Glioblastoma and the Development of Radiosensitizers

**DOI:** 10.3390/cancers12092511

**Published:** 2020-09-03

**Authors:** Md Yousuf Ali, Claudia R. Oliva, Abu Shadat M. Noman, Bryan G. Allen, Prabhat C. Goswami, Yousef Zakharia, Varun Monga, Douglas R. Spitz, John M. Buatti, Corinne E. Griguer

**Affiliations:** 1 Interdisciplinary Graduate Program in Human Toxicology, University of Iowa, Iowa City, IA 52242, USA; ali-mdyousuf@uiowa.edu; 2 Free Radical & Radiation Biology Program, Department of Radiation Oncology, Holden Comprehensive Cancer Center, University of Iowa, Iowa City, IA 52242, USA; claudia-oliva@uiowa.edu (C.R.O.); bryan-allen@uiowa.edu (B.G.A.); prabhat-goswami@uiowa.edu (P.C.G.); douglas-spitz@uiowa.edu (D.R.S.); 3 Department of Radiation Oncology, University of Iowa Hospitals and Clinics, Iowa City, IA 52242, USA; john-buatti@uiowa.edu; 4 Department of Biochemistry and Molecular Biology, The University of Chittagong, Chittagong 4331, Bangladesh; asmnoman.bmb@cu.ac.bd; 5 Department of Pathology, McGill University, Montreal, QC H3A 2B4, Canada; 6 Department of Internal Medicine, University of Iowa, Iowa City, IA 52242, USA; yousef-zakharia@uiowa.edu (Y.Z.); varun-monga@uiowa.edu (V.M.)

**Keywords:** glioblastoma, radioresistance, radiosensitizer

## Abstract

**Simple Summary:**

Numerous mechanisms of glioblastoma (GBM) radioresistance have been identified but have not yet resulted in development of effective radiosensitizer that can increase the efficacy of radiotherapy. In this review, the authors review the mechanisms of GBM radioresistance along with current status of radiation treatment and imaging techniques used in GBM diagnosis and radiotherapy. In addition, they summarize the current GBM radiosensitizers that are being investigated or enrolled in clinical trials. This review emphasizes on the importance of developing an effective radiosensitizers to increase the outcome of GBM radiotherapy. The authors highlight the importance of discovering of novel mechanism(s) of GBM radioresistance that will lead in developing an effective radiosensitizer.

**Abstract:**

Ionizing radiation is a common and effective therapeutic option for the treatment of glioblastoma (GBM). Unfortunately, some GBMs are relatively radioresistant and patients have worse outcomes after radiation treatment. The mechanisms underlying intrinsic radioresistance in GBM has been rigorously investigated over the past several years, but the complex interaction of the cellular molecules and signaling pathways involved in radioresistance remains incompletely defined. A clinically effective radiosensitizer that overcomes radioresistance has yet to be identified. In this review, we discuss the current status of radiation treatment in GBM, including advances in imaging techniques that have facilitated more accurate diagnosis, and the identified mechanisms of GBM radioresistance. In addition, we provide a summary of the candidate GBM radiosensitizers being investigated, including an update of subjects enrolled in clinical trials. Overall, this review highlights the importance of understanding the mechanisms of GBM radioresistance to facilitate the development of effective radiosensitizers.

## 1. Introduction

Glioblastoma (GBM) is the most common adult primary malignant brain tumor and is also the most lethal [1,2]. Median progression-free and overall survival after initial diagnosis are 6.2–7.5 and 14.6–20.5 months, respectively, even with a highly aggressive standard-of-care treatment consisting of maximum safe surgical resection, radiation therapy, and chemotherapy [3,4,5,6,7,8]. In light of this grim prognosis, substantial effort has been invested to improve the overall survival of patients with GBM. However, over the last decade, all preclinical strategies that have shown promise for improving the outcome of GBM treatments have failed to provide an overall survival benefit in large randomized clinical trials [4,5,6,9,10]. The main reason for these failures is attributed to the development of resistance to standard therapeutic options for GBM, which include radiotherapy with concomitant chemotherapy. In particular, the development of adaptive radioresistance has been a major challenge. In the hope of identifying a method to overcome this urgent clinical problem, significant research has focused on defining the molecular mechanisms of adaptive radioresistance in GBM. This review presents a brief synopsis of historic advances in GBM diagnosis and treatment, along with reported findings from pre-clinical studies and the clinical trials of candidate radiosensitizers in GBM.

## 2. History and Current Status of GBM Detection and Imaging Techniques

**Figure 1:**
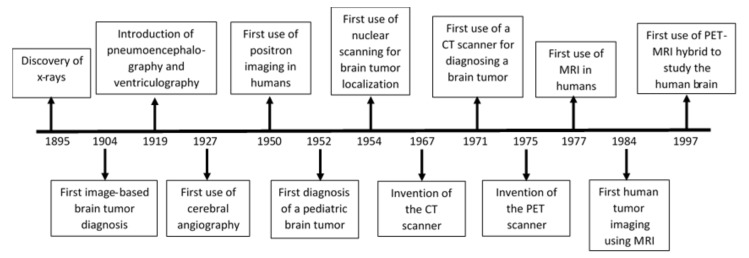
Timeline of important discoveries and events that led to current imaging techniques for detecting glioblastoma (GBM) and other brain tumors. CT, computed tomography; MRI, magnetic resonance imaging; PET, positron emission tomography.

Standard-of-care treatment for GBM includes surgical resection of the tumor, followed by radiotherapy with concomitant daily temozolomide (TMZ) chemotherapy. Successful surgical resection of the GBM tumor and radiotherapy largely depend on proper tumor imaging and diagnosis. The appearance and location of the tumor suggest both the diagnosis and surgical approach, as well as the safety of resection. Diagnostic imaging of GBM and other brain tumors has significantly improved throughout the last century (Figure 1). The first successful image-based diagnosis of any brain tumor was achieved using X-rays in 1904, roughly a decade after the discovery of X-rays by Wilhelm Röentgen in 1895 [11]. In the next few decades, several other techniques for diagnosing brain tumors were developed and used, such as skull radiographs, pneumoencephalography, ventriculography, myelography, and cerebral angiography [11,12,13,14,15,16]. Among these techniques, pneumoencephalography was the first technique that allowed visualization, although indirect, of the living brain. The first pediatric brain tumor was diagnosed and reported in 1952 using this technique [17]. In 1954, the utility of nuclear scanning using radioisotopes for localizing brain tumors was reported [18]. Nuclear scanning was the first noninvasive method available to localize brain tumors and has been used consistently ever since. A new era of neuroimaging-based diagnosis began in 1971, after the invention of the computed tomography (CT) scanner by Sir Godfrey N. Hounsfield. For the next decade, CT was widely used and described as the most accurate technique for diagnosing brain tumors [19,20,21,22]. Beginning in the1980s, however, the popularity of CT for brain tumor diagnosis began to decline as studies started to report better diagnosis clarity with magnetic resonance imaging (MRI). MRI provides vastly improved soft-tissue contrast, high spatial resolution, and rapid widespread availability [23]. The later introduction of spiral or helical CT technology, which allows the array detector to spin continuously around the patient, afforded even greater improvements, including the ability to obtain many more images with far greater speed. The greatest advantage of spiral CT for brain tumor imaging has been the consequent ability to create CT angiograms and conduct time-dependent blood perfusion measurements [24].

Despite the improvements to CT, MRI is the preferred imaging technique to characterize gliomas, with approximately 80% of primary malignant brain tumors characterized by MRI [25]. MRI is more sensitive than CT, as indicated by the better correlation of gross and microscopic autopsy findings with MRI than with CT. Compared with MRI, CT usually provides poorer resolution and underrepresents the size of brain tumors [26]. In 1984, when MRI was first used for brain tumor imaging [23], it was still considered an anatomic imaging method. This characterization changed after the deployment of diffusion-weighted imaging (DWI) and diffusion tensor imaging (DTI) [27]. These advancements were based on the concept that tumors have a higher cellular density than normal tissue, so fluid in the extracellular space of crowded tumor environments will diffuse less readily. Comparison of apparent diffusion coefficients calculated with DWI can distinguish tumor from non-tumor tissue [28,29] and even GBM from central nervous system lymphoma [30,31]. Moreover, dynamic contrast-enhanced (DCE) MRI and dynamic susceptibility (DSC) MRI are two other advanced MRI techniques that can help in monitoring physiological and biological processes in GBM [32,33]. DSC and DCE-MRI are based on modulation and modification of T1 and T2 relaxation time. Although T1 and T2 relaxation are naturally present as signal contrast mechanisms, intrinsic changes in these mechanisms due to disease processes can be quite subtle [34]. Therefore, exogenous contrast agents (CAs), such as gadolinium-based gadopentetate dimeglumine, are sometimes used in clinical oncology MRI studies. DCE-MRI is performed by modulating T1 relaxation time using exogenous CAs. The method is based on the exchange of exogenous CAs between the intravascular compartment and the interstitial tissue. The time course of the diffusion of the contrast agent from the blood pool into tissues through leaky blood vessels is measured to accomplish DCE-MRI. DSC-MRI is based on the drop in the T2 signal after the injection of gadolinium-based CAs and the magnetic susceptibility of the particular tissue [35]. Localized MR spectroscopy imaging (MRSI) is another advanced modality of MRI that depends on the metabolic characteristics of tissue for evaluation of brain tumors. MRSI uses unique spectra originating from nuclei such as proton (1H), phosphorus (31P), and carbon (13C) spectra to measure brain metabolites [36,37]. Conventional MRI provides anatomical information and differences in the morphological structure of the brain tumor. However, anatomical images based on MRI do not provide all the information about molecular changes in response to therapy. MRSI can provide that complimentary information, as it non-invasively maps metabolic profiles and dynamics of the GBM tumor [36,38]. MRSI can perform both steady-state and kinetic analysis of cancer metabolism in vivo and can detect a variety of metabolites [39,40]. Therefore, combining the molecular information provided by MRSI with anatomical information from conventional MRI would provide a better strategy for GBM patient management. MRSI has been reported for use in GBM treatment planning and follow-up of patients after radio- and chemotherapy [41,42,43]. All these advancements in MRI are crucial for detecting the response of patients to radiotherapy and managing GBM radioresistance. Lately, positron emission tomography (PET) has also been used along with MRI to provide additional insight into the biology of gliomas, which can improve planning for surgery and radiotherapy [44,45]. Additional potential imaging information that may impact GBM treatment includes radiomics, the conversion of biomedical images into quantitative data.

Radiomics allows advanced non-invasive assessment of complex imaging features obtained by MRI that may serve as biomarkers [46,47] of disease aggressivity or response. Although these major advances in imaging techniques have substantially improved our ability to diagnose brain tumors, including GBM, overall survival and prognosis for patients with GBM continues to be poor, mostly due to inherent and developed resistance against standard-of-care therapy.

## 3. Treatment Options for GBM/History of GBM Treatment

Despite the growing number of preclinical studies and clinical trials for GBM, current treatment options have not made significant gains in improving patient survival. GBM treatment is particularly challenging because of the primary location, intrinsic heterogeneity, and infiltrating growth pattern of these tumors. Standard-of-care treatment for GBM includes surgical resection of the tumor, followed by radiotherapy with concomitant daily temozolomide (TMZ) chemotherapy followed by additional TMZ therapy. Surgical treatment in GBM aims for maximal surgical resection, thereby improving conditions for complimentary treatments with chemo- and radiotherapy. Extent of resection (EOR) by surgery is an important treatment-related predictor, as more extensive surgical removal is associated with longer life expectancy [48,49,50]. Surgical resection is followed by concurrent TMZ and radiotherapy. TMZ is given in a dose of 75 mg/m^2^/day for six weeks and radiotherapy is given in 30 fractions, totaling 60 Gy, followed by six maintenance cycles of TMZ (150–200 mg/m^2^/day for the first five days of a 28-day cycle) [5,51]. GBM tumors with epigenetic silencing of the *MGMT* (O^6^-methylguanine-DNA methyltransferase) DNA-repair gene by promoter methylation seem to benefit the most from the addition of TMZ [52]. A recent study [53] suggests that dual alkylator therapy with temozolomide and lomustine might improve survival compared with standard temozolomide therapy in patients with newly diagnosed glioblastoma with a MGMT-methylated promoter. Most patients eventually experience tumor recurrence or progression. Recurrent GBM tumors are often resistant to temozolomide. Moreover, standard-of-care treatment for recurrent GBM is not well defined. Recurrent patients can benefit from repeat surgery if a total gross resection is possible [54]. Lomustine is used as a second course of alkylating agent to treat most recurrent GBM patients who are eligible for salvage therapy. Bevacizumab is also given as a single agent in some countries including the USA, but not in the European Union. A combination of bevacizumab and lomustine is considered for treatment of recurrent GBM patients with rapidly progressing disease [55]. However, in most cases, these chemotherapies either in combination or alone have failed to show prolongation of overall survival in recurrent GBM patients [56,57]. Therefore, irrespective of the treatment method, most patients diagnosed with primary GBM die within two years.

Until the 1970s, surgery was the only option to treat glioma, with radiation used only as a palliative treatment. However, the scenario changed when several studies in the late 1970s reported the successful use of radiation in treating malignant gliomas in clinical trials [58,59,60]. Over the next few decades, surgical removal of the tumor, followed by radiotherapy, became the standard-of-care therapy for patients with GBM. In 2005, Stupp and colleagues introduced temozolomide (TMZ), a DNA alkylator, in combination with radiation therapy [3]. Surgical removal of the tumor, followed by concomitant radiotherapy and chemotherapy with TMZ, has remained the standard-of-care treatment for GBM since 2005 [61]. Recently, the application of tumor-treating fields (TTFs), which involves the continuous delivery of low-intensity electric fields alternating at an intermediate frequency, has been viewed by some as a promising cancer treatment. TTF therapy has been shown to improve both progression-free and overall survival in GBM [8]. Despite some initial skepticism, application of TTFs to the shaved head through a transducer connected to a portable device has been reported to be effective in patients with GBM in a randomized clinical trial [62]. Moreover, a variety of molecular targeted therapies have been tried both clinically and pre-clinically, such as leflunomide targeting platelet derived growth factor receptor (PDGFR) [63], erlotinib targeting epidermal growth factor receptor [64], tipifarnib targeting Ras [65], temsirolimus targeting mTOR [66], and enzasturin targeting PKC-β [67]. However, nothing is known of the impact of immunotherapy regimens on mechanisms of radioresistance in GBM.

Not all patients respond to these therapies in a similar way. High genetic and molecular variation in GBM tumors makes it difficult to predict individual responses to specific therapeutics. Thus, it is not surprising that despite all these treatment options, the median survival for GBM patients has not dramatically improved. Tumor recurrence, which is almost inevitable after a median survival of 32–36 weeks, further complicates treatment efforts [68,69,70]. Treatment of these recurrent tumors is exceptionally challenging. Reirradiation and stereotactic radiotherapy have been used to treat recurrent GBM tumors [71,72]; however, these and other salvage options are limited by cumulative toxicity [73]. So far, many clinical trials with different chemotherapeutic and recently immunotherapeutic agents administered as single agents or in combinational therapy have been conducted for recurrent GBM [57,74,75,76,77,78], yet none of these combinations has reliably improved survival, highlighting the urgency to find new GBM treatment options.

## 4. Current Status of Radiation Treatment in GBM and Emergence of Radioresistance

Although most patients with GBM have been treated following the same general protocols over the last decade, radiation therapy has changed substantially over this period as a result of better instrumentation and improvements in imaging technology [79]. These changes include an upgrade in radiotherapy technology from 2-dimensional whole-brain radiotherapy to 3-dimensionional conformal radiotherapy, and more recently to intensity-modulated radiation therapy (IMRT) and volumetric arc radiation therapy (VMAT) [80,81]. IMRT techniques limit the exposure of normal tissues to radiation by delivering non-uniform, computationally optimized radiation to the tumor. In addition, fractionated stereotactic radiation has been suggested and used in several clinical trials for patients with recurrent GBM. Stereotactic guidance further improves the accuracy of treatment delivery to a radiographically identified target. With fractionation, the total dose of radiation is split into many smaller fractions and administered over a span of several weeks, which improves the radiobiological impact on tumors versus normal tissues, which repair damage more quickly. An approach known as hypofractionated radiotherapy allows the total dose of radiation to be split into larger doses, thus fewer fractions, and administered over a shorter period, which may improve convenience, although it does not appear to improve overall survival of patients [73,82,83,84]. A limited number of studies combining advanced image-based targeting of GBM with dose escalation suggest a small benefit in outcomes [85,86,87]. Thus, despite major advances in radiation technology, the overall outcome of radiotherapy in GBM remains far from optimal, as tumors are inherently resistant and develop increased resistance to radiation, especially upon recurrence.

## 5. Mechanisms of GBM Radioresistance

**Figure 2:**
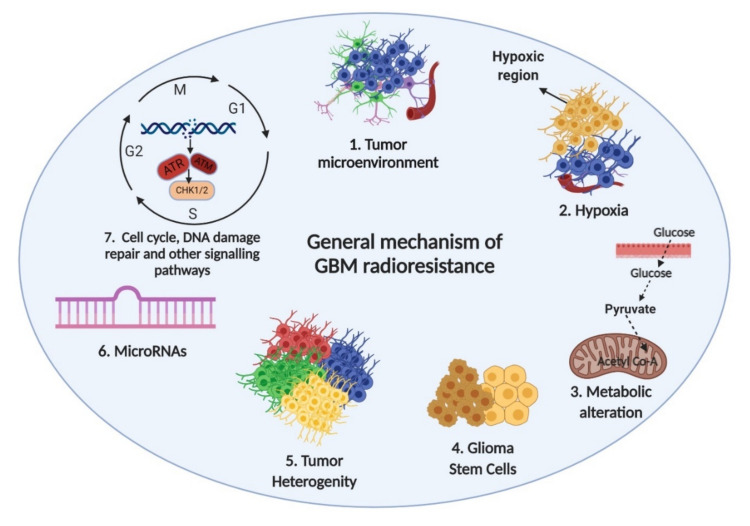
Identified mechanisms of GBM radioresistance. Tumor microenvironment, hypoxia, metabolic alterations, glioma stem cells, tumor heterogeneity, microRNAs, and alteration in cell cycle regulation and DNA damage repair are the most well-defined factors involved in GBM radioresistance. Created with BioRender (Science Suite Inc., Toronto, ON, Canada).

Over the years, many studies have been devoted to elucidating the mechanism of GBM radioresistance. So far, a variety of mechanisms have been implicated to explain GBM radioresistance. All these previously reported mechanisms have identified several key factors, including tumor microenvironment, hypoxia, metabolic alteration, glioma stem cells, tumor heterogeneity, microRNAs, cell cycle, and DNA damage and repair (Figure 2), that contribute to the development of GBM radioresistance. These underlying mechanisms of GBM radioresistance have been discussed in detail in the following subsections.

### 5.1. Tumor Microenvironment

GBM tumor microenvironment plays a major role in tumorigenesis and progression of GBM. Cellular composition of the GBM tumor microenvironment includes non-neoplastic stromal cells, normal and reactive astrocytes, fibroblasts, extracellular and vascular pericytes, glioma stem cells, and immune cells [88,89]. The tumor microenvironment also includes different biomolecules produced by all cell types within the tumor to support its growth and progression. All these complex networks of various cells and biomolecules in the tumor microenvironment contribute to the radiation response [90]. Several studies have reported that glioma cells irridiated in vivo in xenograft models show resistance to radiation, whereas same cells grown in vitro exhibit susceptibility to radiation, thus indicating the role of the tumor microenvironment in vivo in developing radioresistance [91,92]. The GBM microenvironment is anatomically compartmentalized in what is referred to as tumor niches, where the signaling arising in stromal and tumor cells converges and regulates tumor progression and proliferation [93,94,95]. Glioma stem cells also reside in these microenviroment niches. Recent evidence suggests that microenvironment niches provide glioma stem cells with a variety of mechanisms to obstruct chemo- and radiotherapies, thus developing resistance [96].

### 5.2. Hypoxia

Hypoxia is common in solid tumors because the rapid tumor growth outpaces the growth of blood vessels, preventing the homogenous diffusion of oxygen to all tumor regions [97]. GBM tumors contain hypoxic regions detected by MRI and microscopic analysis [98], and hypoxia-inducible factors (HIFs) have been shown to contribute critically to GBM tumorigenesis by regulating the tumorigenic capacity of glioma stem cells [99]. In addition, it was reported in the middle of the last century that oxygen concentration influences the response of mammalian cells to radiation [100]. The majority of DNA damage caused by conventional radiotherapy in normoxic conditions is mediated by reactive oxygen species (ROS) such as O_2_^−^, H_2_O_2_, and OH. However, the free radical-generated oxidative stress-inducing capacity of radiotherapy decreases in hypoxic conditions, so it is not surprising that hypoxia leads to the development of radioresistance. Marampon and colleagues reported that regulation of the functional interplay among extracellular signal-related kinases (ERKs), DNA-dependent protein kinase catalytic subunit (DNA-PKcs), and HIF1-α mediated by hypoxia causes radioresistance in GBM [101]. Upon activation by hypoxia, HIF2-α was shown to activate OCT4, a stem cell transcription factor. Upon activation, OCT4 regulates the self-renewal and differentiation of stem cells. Thus, hypoxia can induce radioresistance by increasing stemness in glioma cell populations [102,103,104,105]. Poorly structured blood vessel networks can also result in irregular and fluctuating tumor tissue perfusion. These fluctuations lead to periods of poor and better oxygenation, exposing cells to periods of hypoxia followed by periods of reoxygenation in a cyclic manner [106]. This phenomenon, known as cycling hypoxia, has been reported to induce GBM radioresistance by triggering a substantial increase in HIF1-α activity [107]. Investigators are exploring a variety of mechanisms to minimize hypoxia and reduce radioresistance in GBM. For example, improving intratumoral oxygenation has been reported to increase glioma radiosensitivity in vitro and in vivo. Tracing and applying increasing doses of radiation in hypoxic regions is also being investigated [108,109]. However, more studies are needed to target GBM hypoxia to improve the response to radiotherapy in patients.

### 5.3. Metabolic Alteration

Reprogramming of cellular energetics, or metabolic alteration, is a hallmark of cancer [110] and has an important role in the progression of GBM and other brain tumors. The modification of metabolism and mitochondrial bioenergetics detected in GBM cells fuels survival, proliferation, and invasion. Emerging reports suggest that metabolic alteration also mediates resistance to standard-of-care therapies in GBM [111,112,113,114,115,116,117,118,119]. In particular, high rates of glycolysis have been correlated with GBM radioresistance, and inhibition of the glycolytic pathway has been shown to reduce this resistance in vitro and in vivo [120,121]. The reductant NADPH is a major source of electrons for most cellular antioxidant systems mediated by glutathione and thioredoxin, playing a critical role in redox metabolism and facilitating survival against numerous pro-oxidants, such as radiation. In IDH1wtGBM, the wild-type IDH1 mediates the production of NADPH in response to radiation, facilitating radioresistance. Conversely, knockdown of wild-type IDH1 has been reported to reduce the level of NADPH, making GBM cells radiosensitive in vitro and in vivo [122,123]. High tumor expression of the ATPase family, AAA domain-containing 3A (ATAD3A), a nuclear DNA-encoded mitochondrial protein involved in maintaining mitochondrial functions, and communication between the endoplasmic reticulum (ER) and mitochondria, have been shown to correlate with the development of GBM radioresistance [124]. Moreover, it has been reported that mitochondrial ATP-sensitive potassium channels are overexpressed in glioma and control glioma radioresistance by regulating ROS-induced ERK activation [124]. Knockdown of *TP53*-induced glycolysis and apoptosis regulator (TIGAR) has been shown to radiosensitize glioma cells to radiation [125]. TIGAR, an early target of p53, can increase the level of NADPH, redirecting glucose into the pentose phosphate pathway. Increased NADPH helps cells to deal with redox stress. Therefore, it is possible that TIGAR induces radioresistance by helping GBM cells to handle radiation-induced redox stress. Altogether, numerous studies show a correlation between metabolic alterations and GBM radioresistance, although the direct mechanistic link between metabolic reprogramming and GBM radioresistance remains to be elucidated.

### 5.4. Glioma Stem Cells

**Figure 3:**
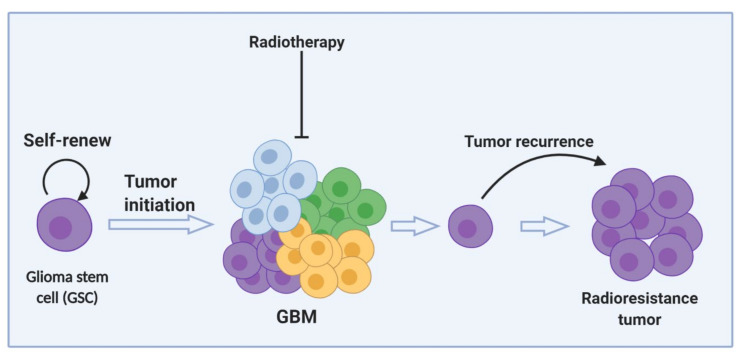
GSCs can self-renew, initiate tumors, and survive radiotherapy. The cells that survive radiotherapy can give rise to a population of cells that are resistant to radiation. Created with BioRender (Science Suite Inc., Toronto, Ontario, Canada).

In recent years, cancer stem cells (CSCs), also known as tumor initiating cells, have been extensively reported in different cancer types. CSCs are a subpopulation of cells within a tumor mass that have the ability to self-renew and differentiate into diverse types of tumor cells [126,127]. Several studies have demonstrated the existence of self-renewing tumorigenic cells in GBM and other gliomas that show multilineage differentiation potential and stem cell marker expression, and which are thus referred to as glioma stem cells [128,129,130,131,132]. In addition, glioma stem cells have been shown to propagate as therapy-resistant cells [133,134,135], as shown in Figure 3. Glioma initiating cells (GICs) are resistant to radiation and are directly correlated with patients’ outcomes [136]. GICs can be characterized by the expression of a group of markers such as SOX2, OCT4, NANOG, OLIG2, NESTIN, ID1, CD133, CD15, CD44, and A2B5 [137,138,139,140,141,142,143,144,145]. The fraction of glioma cells expressing CD133, a marker for both neural and GICs [130], increases after irradiation. CD133-positive GICs preferentially activate DNA damage checkpoint proteins such as Chk1 and Chk2 in response to radiation, carrying out the repair of radiation-induced DNA damage more effectively than CD133–negative cells. Thus, an enhanced DNA damage repair capacity likely underlies, at least in part, the radioresistance of CD133-positive GICs [134]. It was also shown that GICs become radioresistant through the overexpression of proliferating cell nuclear antigen (PCNA)-associated factor (PAF). [146]. PAF is a DNA damage-regulated factor that controls the accessibility of DNA translesion synthesis (TLS) enzymes to PCNA, thereby facilitating DNA damage bypass [147]. After irradiation of GICs, PAF associates with PCNA to release TLS Pol η, resulting in restoration of error-free DNA synthesis and, in turn, glioma stem cell proliferation and radioresistance [146]. Moreover, high expression of cathepsin L, a lysosomal endopeptidase enzyme, mediates radioresistance in GICs. Interestingly, knockdown of cathepsin L in patient-derived GICs led to decreased expression of CD133 and reduced phosphorylation of DNA damage checkpoint proteins, restoring radiosensitivity [148]. However, further studies are needed to determine how cathepsin L promotes these effects. Overall, these studies show that the presence of GICs in GBM might play a critical role in promoting radioresistance.

### 5.5. GBM Tumor Heterogeneity

Tumor heterogeneity is characterized by the presence of different cell populations or clones having distinct genetic or molecular profiles within a tumor or among different individual tumors originating from the same tumor. Intertumoral heterogeneity is defined by distinct genetic alterations present in individual tumors originating in the same organ, whereas intratumoral heterogeneity is characterized by distinct genetic alterations within the same tumor [149,150]. Intratumoral heterogeneity is further complicated by the presence of different cell types [151]. GICs residing in the GBM microenvironment niche play a major role in tumor heterogeneity. GSCs are characterized by their ability to regenerate, whereas GBM initiating cells are a subpopulation of GSCs that are CD133+ and are capable of tumor initiation in orthotopic mouse models [152]. GSCs and GBM initiating cells have been shown to contribute to GBM radioresistance through increased activation of DNA damage checkpoint pathways and intrinsic hyperactivation of PI3/Akt and PTEN pathways [134,153]. The differences in molecular and genetic signatures of these different cells within a single tumor cause differential responses to radiotherapy among specific cell populations. Upon treatment with radiation, the radioresistant populations eventually become dominant, leading to an overall increase in tumor resistance [154].

Intratumoral heterogeneity creates a major challenge in the treatment of GBM. Heterogeneity has been detected among tumors from different patients, yet molecular analysis of patient-derived GBM tissue has shown genetic diversity within regions of individual tumors as well [155,156,157]. Single-cell RNA sequencing and integrated genomic analysis of GBM tissues have shown unique transcriptional programs within individual tumors and clinically relevant subtypes [150,158]. Moreover, molecular analysis has revealed multiple cellular subclones within in a single GBM tumor. Genomic analysis of GBM tumors has identified four major subtypes based on gene expression patterns, namely classical, pro-neural, neural, and mesenchymal. Alterations in the expression of *EGFR, NF1,* and *PDGFRA/IDH1* genes identify classical, mesenchymal, and pro-neural subtypes, respectively, whereas the neural subtype is defined by the expression of several neural markers such as *NEFL, GABRA1, SYT1*, and *SLC12A5* [159]. However, subsequent studies have redefined the transcriptional subtypes of GBM into three clinically relevant classes, designated as proneural, mesenchymal, and classical [160]. GBM tumors also vary in the status of several other genes, with such variety including differences in isocitrate dehydrogenase (*IDH)* mutation and O6-methylguanine-DNA methyl transferase (*MGMT)* promoter methylation [161,162]. IDH is an enzyme that catalyzes the decarboxylation of isocitrate to α-ketoglutarate. IDHs have three isoforms, namely IDH1, 2, and 3. The majority of *IDH* mutations in GBM involve R132 of *IDH1* [163,164]. The R132 *IDH1* mutation is more common in secondary GBM than in primary GBM [161,165]. *IDH1* mutation has a better prognosis, although exceptions have also been reported [166,167]. *IDH1* mutations have been reported to radiosensitize glioma cells by epigenetic downregulation of TIGAR. Moreover, *IDH1* silencing can improve the response of GBM cells to radiation by reducing the level of NADPH [123,168]. *MGMT* encodes for a DNA repair enzyme that repairs and detoxifies TMZ-induced DNA damage [169]. A combination of *IDH1* mutation and MGMT methylation has been reported to better predict the outcome of TMZ and radiotherapy than either *IDH1* or MGMT alone [170,171]. As the combination of MGMT methylation and *IDH1* show a correlation with better patient outcomes following radiotherapy, it remains to be investigated if these two mechanisms can be targeted in radioresistant GBM cells.

### 5.6. MicroRNAs

MicroRNAs are small non-coding RNAs that usually inhibit gene expression at the posttranscriptional level. Altered expression of several microRNAs has been reported in different cancers [172,173,174,175], and the role of microRNAs in GBM has been studied extensively [176,177]. A literature survey conducted in 2013 reported that around 235 microRNAs are overexpressed and 95 are downregulated in GBM, compared with normal brain tissue [178]. Notably, microRNAs have been shown to effectively regulate radiation-related signal transduction pathways in GBM, and many studies have reported that the radiosensitivity of GBM can be altered by targeting these microRNAs. For example, miR-124 was found to increase the radiosensitivity of glioma cells by targeting and inhibiting CDK4 [179,180]. In addition, Patryk and colleagues have shown that overexpression of miR-1 and miR-221/222 confer radioresistance in GBM cells by regulating AKT, independently of PTEN status. Upon activation by miR-221/222 after irradiation, AKT modulates DNA-PKcs expression to enhance DNA damage repair (DDR) activity and thereby promote radioresistance [181]. Another study reported that miR-1, miR-125a, miR-150, and miR-425 induce radioresistance in GBM through upregulation of the cell cycle checkpoint response [182]. Thus, these studies show that different microRNAs can regulate GBM radioresistance by modulating Akt signaling, cell cycle checkpoint responses, and DDR activity.

### 5.7. Cell Cycle, DNA Repair and Other Signaling Pathways

Several studies have reported the role of the DNA repair pathways in GBM radioresistance. Marampon and colleagues reported that histone deacetylase (HDAC)-4 and -6 promote radioresistance in GBM by inducing double strand break (DSB) repair [183]. Overexpression of α-6 integrin also causes radioresistance in GBM by increasing the efficiency of DDR [184]. Furthermore, overexpression of EGFR and EGFRvIII cause radioresistance in GBM by activating both homologous recombination and nonhomologous end joining. EGFRvIII has been shown to activate a key enzyme, DNA-PKcs, involved in DSB repair [185,186]. BMI1, a component of the polycomb repressive complex 1 (PRC1), is associated with the proliferation of high-grade gliomas and other cancer types [187,188,189,190]. BMI1 was also reported to confer radioresistance to GBM by recruiting DDR machinery [191].

GBM is a heterogenetic tumor that often harbors anomalies in a variety of signaling pathways. Alterations in several molecular and signaling pathways have been shown to be involved in inducing radioresistance in GBM [192]. One of the pathways reported to be intricately involved in this resistance is the Notch signaling pathway. This signaling pathway is important in the maintenance of a variety of cells, including neural stem cells, and is known to play an important role in cancer stem cells [193,194,195,196]. Inhibition of Notch 1 and 2 restores radiosensitivity in glioma stem cells, and Notch has been reported to induce radioresistance in GBM through regulation of the PI3-kinase/Akt pathway [197].

In general, the PI3-kinase/Akt signaling pathway is involved in numerous important cellular functions, including cell proliferation, migration, differentiation, metabolism, and apoptosis [198]. Moreover, abnormal activation of the PI3-kinase/Akt pathway is detected in multiple cancer types, including GBM, and is associated with poor prognosis and survival in patients [199]. In GBM, the increase in expression and activity of AKT contributes to tumor progression, recurrence, and radioresistance. Radiation activates Akt in GBM and thereby contributes to the development of radioresistance [200]. Akt has also been shown to be correlated with poor progression-free and overall survival of GBM patients [201,202,203]. Activation of AKT can enhance DNA damage repair (DDR) by promoting γ-H2AX foci resolution in irradiated glioma cells [186], whereas downregulation of AKT facilitates unrepairable DNA double strand breaks (DSB) in irradiated U251 glioma cells [204,205]. In addition, the transmembrane protein leucine-rich repeats and immunoglobin-like domains protein 1 (LRIG1) has been reported to alter GBM radioresistance by modulating the Akt pathway [206]. LRIG1 is expressed in several human tissues and organs and is described as a tumor suppressor [207]. Irradiation causes downregulation of LRIG1 in radioresistant U251R cells [206]. Overexpression of LRIG1 in U251R cells significantly reduced EGFR signaling and AKT phosphorylation, increasing DNA damage and susceptibility to radiation [206], indicating that downregulation of LRIG1 contributes to radioresistance. Expression of *PTEN,* an important gene in the PI3-kinase/Akt pathway, is also frequently altered in GBM [208]. Loss or mutation of *PTEN* leads to activation of Akt, resulting in resistance to radiotherapy. Depletion of *PTEN* has also been shown to sensitize tumor cells to therapies that induce DNA damage, such as radiation [209]. A recent study reported that pharmaceutical inhibition of PTEN phosphorylation at tyrosine 240 sensitizes GBM cells to radiation by attenuating DNA damage repair mediated by nuclear PTEN [210]. Together, these studies indicate that overactivation of Akt signaling promotes GBM radioresistance by modulating DDR and reducing radiation-induced DNA DSBs.

The tumor suppressor p53 is one of most frequently deregulated genes in human cancer and is positioned in the center of the regulatory network controlling cell proliferation, survival, and genome integrity [211]. Around 40%–50% of GBMs have p53 mutations [212,213], and a lack of p53-mediated apoptosis could be a factor in therapy resistance in GBM. Indeed, it has been reported that the failure of p53 to induce p21^BAX^ expression causes radioresistance in GBM-derived cells [214].

Constitutive activation of the JAK/STAT pathway is also common in many cancers. STAT3 is a redox-sensitive transcription factor that is required for the maintenance of stemness in GBM cells [215,216]. In GBM cells, irradiation promotes the nuclear translocation and activation of STAT3, promoting malignancy, and STAT3 activation is high in CD133-positive radioresistant GBM cells and recurrent tumors [217,218]. However, inhibition of STAT3 activity triggers the activation of ERK1/2, which allows GBM cells to survive radiotherapy. Therefore, dual inhibition of STAT3 and ERK1/2 is necessary to sensitize glioma cells to radiation [219]. Another transcription factor, forkhead box protein M1 (FOXM1), which is vital for cell proliferation, cell cycle progression, tissue homeostasis, and DNA damage repair, has been shown to regulate metastasis in different cancers [220,221]. In GBM, high tumor expression of FOXM1 is associated with poor prognosis [222]. Furthermore, in irradiated GBM cells, FOXM1 was shown to mediate radioresistance in a manner that involves direct interaction with STAT3 and is dependent on STAT3 activation [223]. Finally, inhibition of suppressors of cytokine signaling (SOCS), which can regulate JAK/STAT signaling transduction, has been shown to increase radioresistance in glioma cells [224]. In particular, *SOCS3* has been implicated in GBM radioresistance, and methylation of the *SOCS3* promoter may be associated with poor prognosis in patients with GBM [225]. Thus, radiation-induced inhibition of SOCS proteins in glioma cells may activate the JAK/STAT pathway, promoting radioresistance [224]. Overall, these studies show that the JAK/STAT pathway has a major role to play in GBM radioresistance.

The Wnt signaling pathway, best known for critically controlling neural patterning and organ development, has long been described as an important contributor to CSC maintenance in various cancers, including GBM [226,227]. Overexpression of Wnt/β-catenin has been correlated with GBM aggressiveness and poor prognosis of patients [228,229]. Activation of Wnt signaling has also been shown to confer resistance to radiation. For example, multiple Wnt signaling-related genes, including *APC, FZD1, LEF1, TCF4,* and *WISP1*, are overexpressed in radioresistant GBM cells [230]. Moreover, inhibition of the Wnt/ β-catenin pathway restored radiosensitivity in GBM cells displaying adaptive radioresistance [231]. Upon activation, β-catenin translocates to and accumulates in the nucleus, resulting in activation of β-catenin target genes such *MMP-2* and *MMP-9*. Irradiation was shown to mediate these effects in glioma cells, and the activation of *MMP-2* and *MMP-9* after irradiation induced tumor spreading and invasion [232,233]. Although the above studies show correlation of Wnt/β-catenin pathway activation with GBM radioresistance, more research is needed to elucidate the specific mechanisms by which the Wnt pathway promotes this radioresistance.

## 6. Radiosensitizers in GBM and Other Cancer Treatment

Radiotherapy is still the most common treatment option across many tumor types. Around 50% of all cancer patients receive radiation during the course of their treatment, which constitutes 40% of all curative treatments for cancer [234,235,236]. Improved technologies and knowledge about radiation treatment methods have increased the use of radiation therapy. However, there is still a wide range of obstacles and challenges, which have already been discussed in this paper. These challenges, such as the presence of CSCs, tumor heterogeneity, and metabolic alterations, make it difficult to use radiotherapy alone to cure tumors, not only in GBM but in other cancers as well [237,238,239]. In this regard, the use of radiosensitizers has been described as an excellent option for making radiotherapy more effective without increasing the dose of radiation, which may then be detrimental to normal tissues [240,241,242]. Radiosensitizers increase cell sensitivity to radiotherapy by altering the activity of cell factors that modulate the deleterious effects of radiation. Mechanisms of radiosensitization involve inhibiting intracellular thiols [243,244], creating cytotoxic substances [245], inhibiting repair biomolecules [246], and mimicking the electrophilic activity of oxygen [247,248]. The radiosensitizing effects of these mechanisms were related mainly to effects on the DDR pathway induced by radiotherapy. However, over time, the use of radiosensitizers has become a multifaceted approach [249,250]. Some established chemotherapeutic agents have been used as radiosensitizers and have been reported to successfully enhance the efficacy of radiotherapy in clinical trials for different cancers [251,252]. For example, the chemotherapeutic agent gemcitabine has been shown to be an effective radiosensitizer in the treatment of many cancers, such as breast, ovarian, non-small cell lung, pancreatic, and bladder cancers [253,254,255]. In addition, the small molecule inhibitor of c-MET, crizotinib, has been shown to enhance the radiosensitivity of KRAS-mutant colorectal cancers that are resistant to cetuximab [256]. Pretreatment of breast and lung cancer cells with a novel estrone analog also increases sensitivity to radiation [257].

As discussed earlier, one of the main reasons for radioresistance in GBM and other solid tumors is the presence of hypoxic regions within the tumor. Therefore, oxygen-mimicking compounds have been investigated as potential radiosensitizers in different cancers [258,259,260]. In particular, compounds containing a nitro group that has the same electron affinity as oxygen have been described to have radiosensitizing effects [242,261,262,263,264]. Furthermore, oxygen carriers and agents that can produce oxygen, such as hydrogen peroxide, have also been described as potential radiosensitizers [265,266]. However, insufficient and poorly formed blood vessels in the tumor microenvironment make it difficult to increase tumor oxygenation therapeutically [267]. Therefore, the alternative approach of reducing mitochondrial respiration has been investigated as a method for increasing oxygenation in hypoxic tumor regions [268]. This approach, termed metabolic radiosensitization, reduces the cellular metabolic demand for oxygen by reducing mitochondrial oxidative metabolism. In this regard, Benaj and colleagues have shown that papaverine, an inhibitor of mitochondrial complex I, increases tumor oxygenation and thus sensitizes the cells of hypoxic lung and breast tumors—but not healthy, normoxic tissues—to radiation in mouse models [269].

As radiotherapy is an integral component of the standard-of-care therapy for GBM, the use of radiosensitizers has been promoted as a potential treatment option for GBM as well [270,271], and many chemotherapeutic agents have been investigated [272,273,274,275]. However, most potential GBM radiosensitizers have not progressed to clinical trials due to a lack of promising preclinical data (Table 1).

Of the proposed radiosensitizers that were effective in preclinical studies and thus evaluated to phase I/II clinical trials (Table 2), most failed to improve progression-free and overall survival and did not progress to phase III. However, we are still awaiting the results of phase II clinical trials for some agents, as shown in Table 2. Therefore, continued research into the mechanisms of radioresistance is needed to identify novel candidate radiosensitizers.

## 7. Conclusions

It is clear from the literature that GBM remains a very deadly cancer, despite the myriad research efforts and clinical trials with agents designed to improve the treatment outcome. Moreover, in patients with newly diagnosed or recurrent GBM, outcomes with radiotherapy have not improved for years. Radiosensitizers have been considered and remain a viable option for improving the outcome of therapy in GBM but have not yet achieved this potential. Overall, more research is necessary to fully understand the mechanisms of GBM radioresistance and improve the outcomes of patients with this deadly disease.

## Figures and Tables

**Table 1:** List of drugs investigated as potential radiosensitizers for GBM treatment.

Name of the Radiosensitizers	Effect	References
Gemcitabine	Initiates DNA damage by incorporating gemcitabine triphosphate, an active metabolite of gemcitabine, instead of nucleotide deoxycytidine triphosphate (dCTP)	[272,276]
Gö6976	Protein kinase inhibitor	[277]
Talazoparib	PARP inhibitor	[278]
MEK162	MAPK inhibitor	[279]
Erlotinib	EGFR inhibitor	[280]
Everolimus	mTOR inhibitor	[281]
Valproate	HDAC	[282]
Vorinostat	HDAC inhibitor	[283]
Vandetanib	VEGFR2 inhibitor	[284]
Enzastaurin	Protein Kinase C (PKC) inhibitor	[285]
Talampanel	alpha-amino-3-hydroxy-5-methyl-4-isoxazolepropionic acid (AMPA) receptor antagonist	[286]
TMZ	Alkylates/methylates DNA at N-7 or O-6 positions of guanine residue	[287]
Bortezomib	Proteasome inhibitor	[288]
Resveratrol	STAT3 inhibitor	[218]
Veliparib	PARP inhibitor	[289]
Adavosertib	WEE1 inhibitor	[290]
Chloroquine	Inhibits autophagy and induces apoptosis	[291,292]
Ascorbate	Pro-oxidant	[293]
RRx-001	Macrophage-stimulating agent	[294]
Motexafin gadolinium	Inhibits thioredoxin reductase and ribonucleotide reductase	[295]
NVX-108	Carries oxygen to the hypoxic tissue	[296]
Trans sodium crocetinate	Enhances oxygen levels in hypoxic tissue	[297]
Arsenic trioxide	Activates apoptosis and autophagy	[298,299,300]
Sulfasalazine	Inhibits cystine uptake	[301]
Palbociclib	CDK inhibitor	[302]
KU - 55933	ATM inhibitor	[303]
AZD1390	ATM inhibitor	[304]

**Table 2:** List of current and previous clinical trials of radiosensitizers for GBM treatment.

Study ID	Phase	Diagnosis	Treatment	Outcomes
NCT01752491	I	GBM	Ascorbate, TMZ, and radiotherapy	No dose-limiting toxicities [293]
NCT01465347	I & II	GBM	Trans sodium crocetinate (TSC), TMZ, and radiotherapy	No adverse effects. Suggests radiotherapy and TSC combination is beneficial for GBM treatment. No significant difference in overall survival [297].
NCT00185861	I	Recurrent malignant glioma	Arsenic trioxide (ATO) and stereotactic radiotherapy	ATO and fractionated stereotactic radiotherapy is well-tolerated [298]
NCT04205357	I	Recurrent GBM	Sulfasalazine and stereotactic radiotherapy	Study ongoing, recruiting patients
NCT02871843	I	GBM, oligodendroglioma, anaplastic oligodendroglioma	RRx-001, TMZ, and radiotherapy	Study ongoing
NCT00302159	II	High-grade gliomas	Valproic acid (VPA), TMZ, and radiotherapy	No adverse effects; VPA in combination with TMZ and radiotherapy can improve outcome [282]
NCT00305864	I & II	GBM	Motexafin gadolinium, TMZ, and radiotherapy	No adverse effects; no significant improvement in overall survival [295]
NCT03862430	II	GBM	NVX-108, TMZ, and radiotherapy	Study ongoing
NCT03672721	I & II	GBM	Carboplatin and radiotherapy	Study ongoing
NCT02378532	I	GBM	Chloroquine, TMZ, and radiotherapy	No adverse effects reported [305]
NCT02432417	II	GBM	Chloroquine, TMZ, and radiotherapy	Study ongoing
NCT01849146	I	Newly diagnosed and recurrent GBM	Adavosertib, TMZ, and radiotherapy	Study ongoing
NCT03423628	I	GBM	AZD1390 and Radiotherapy	Study ongoing
